# Mining Important Herb Combinations of Traditional Chinese Medicine against Hypertension Based on the Symptom-Herb Network Combined with Network Pharmacology

**DOI:** 10.1155/2022/5850899

**Published:** 2022-03-22

**Authors:** Zhenhai Sun, Yunsheng Xu, Wenrong An, Siling Bi, Sai Xu, Rui Zhang, Mingyang Cong, Shouqiang Chen

**Affiliations:** ^1^Shandong University of Traditional Chinese Medicine, Jinan, China; ^2^The Second Hospital, Shandong University of Traditional Chinese Medicine, Jinan, China

## Abstract

Although data mining methods are extensively used in the rule analysis of famous old traditional Chinese medicine (TCM) experts' prescriptions for the treatment of hypertension, most of them only mine the association between herbs and herbs, ignoring the importance of symptoms in the disease. This study collected 439 cases of hypertension treated by famous old TCM experts from the FangNet platform. Using the structure network algorithm, the symptom-herb network was constructed, which redefined the importance of herb in disease. Based on the network, 21 driver herbs, 76 herb pairs, and 41 symptom-herb associations were mined. Finally, the basic prescription composed of Gouteng (Uncariae Ramulus cum Uncis), Huanglian (Coptidis Rhizoma), Chuanxiong (Chuanxiong Rhizoma), Gegen (Puerariae Lobatae Radix), Danggui (Angelicae Sinensis Radix), and Huangqin (Scutellariae Radix) was found. These herbs are the most significant among all herbs, and they have a potential correlation with each other. To further verify the rationality of the data mining results, we adopted the network pharmacology method. Network pharmacological analysis shows that the five core targets in the basic prescription include IL6, VEGFA, TNF, TP53, and EGF, which link 10 significant active compounds and 7 important KEGG pathways. It was predicted that anti-inflammatory, antioxidant, vascular endothelial protection, emotion regulation, and ion channel intervention might be the main mechanisms of the basic prescription against hypertension. This study reveals the prescription rule of famous old TCM experts for treating hypertension from a new perspective, which provides a new approach to inherit the academic experience of famous old TCM experts and develop new drugs.

## 1. Introduction

As a worldwide public health problem, the incidence rate and mortality rate of hypertension are increasing year after year [1]. It is estimated that 150 million people will unfortunately suffer from hypertension by 2025 [2]. The long-term rise of blood pressure will damage the target organs such as the kidney, brain, and heart, causing a variety of complications including chronic renal failure, dementia, coronary heart disease, and so on [3]. These complications are also a crucial reason for the increase of mortality [4].

At present, based on the different pathological mechanism of hypertension, its therapeutic drugs mainly include diuretics, *β* blockers, calcium channel blockers, angiotensin-converting enzyme inhibitors, and angiotensin II receptor blockers [5]. Although these drugs have obvious antihypertensive effects, their mechanism of action is single, and long-term usage may cause various adverse events, such as angioedema and cough, affecting patients' compliance [6–9]. Therefore, the treatment of hypertension should not only regulate the level of blood pressure but also consider the protection of target organs and reduce the side effects of drugs. In the above context, the treatment of hypertension with multitarget or multicomponent drugs has increasingly become the focus of attention [10].

TCM has been treating hypertension for more than 2000 years in China. TCM has a unique understanding of hypertension due to long-term clinical practice. In TCM theory, hypertension mainly falls into the category of “dizziness” and “headache.” Herb is one of the essential means of TCM against hypertension, which has the characteristics of multitarget, multicomponent, and multipathway for treating diseases. Herb treatment of hypertension has the advantages of synergistic lowering of blood pressure, improving symptoms, improving metabolism, and protecting target organs [11]. It has become a hot topic to try developing new drugs for the treatment of hypertension from the hidden information of herbs.

In the clinical practice of TCM, the formula composed of at least two or more herbs is an important form in which herbs can play a therapeutic effect [12]. These formulas can reflect the academic experience of TCM experts [13]. With the advent of the era of big data, a large number of effective clinical cases of famous Chinese medicine experts have been preserved, in which the formulas and symptoms hide rich information [14].

Currently, data mining methods such as association rules and cluster analysis are widely used in the field of TCM, including condition, diagnosis, and formula, which makes the hidden information in cases interpreted at multiple levels [15]. Based on biomolecular networks, network pharmacology studies the relationship among herbs, components, targets, and diseases from the perspective of interconnection, which meets the needs of systematic treatment of complex diseases. Its core thought has something in common with the holistic view of TCM [16, 17].

**Figure 1:**
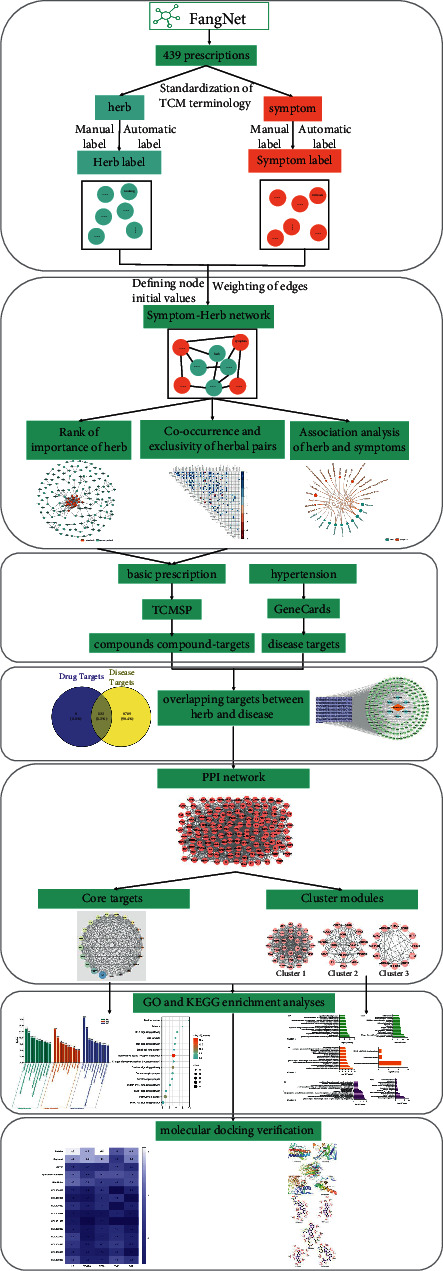
The workflow of this paper.

In this study, we used data mining methods based on structure network algorithm with network pharmacology technology to mine and preliminarily verify the potential information in the treatment of hypertension cases by famous old TCM experts, which may be an efficient way to inherit the academic experience of famous old TCM experts and develop new drugs. The workflow of this paper is illustrated in Figure 1.

## 2. Materials and Methods

### 2.1. Source of Clinical Prescription

Related cases were collected from FangNet (https://fangnet.org), which is a platform for collecting the medication experience of famous old TCM experts. A new collection named “Hypertension” was established in the prescription collection module. In the prescription module, we can search for cases with a disease named hypertension and select them to copy to the new collection. A total of 439 relevant cases were finally retrieved.

#### 2.1.1. Inclusion Criteria

① Clinical studies of TCM prescriptions in treating hypertension are included. ② Cases should contain clear information about symptoms and herbs. ③ Cases with clear treatment effect were included in this study. ④ Repeated prescriptions were included only once.

#### 2.1.2. Exclusion Criteria

① Incomplete information among cases. ② No obvious therapeutic effect of the patients. ③ Repeated cases.

### 2.2. Standardization of TCM Terminology

FangNet has established a symptom/herb semantic repository by integrating SymMap [18], a comprehensive symptom database of TCM, with Chinese Pharmacopoeia 2020 Edition [19]. It contains standardized nouns for 1,717 symptoms and 618 herbs. Relevant symptoms and herbs in cases can be automatically extracted and labeled to terms in the semantic repository if they can match. To maximize the accuracy of the analytical results, herbs and symptoms that could not be matched by the symptom/herb semantic repository were manually labeled and standardized with reference to the WHO International Standard Terminology on Traditional Medicine in The Western Pacific Region. After extracting the labels of symptoms and herbs for all cases and clicking on “View details,” we check all labels errorless and then begin the analysis.

### 2.3. Symptom-Herb Association Network Construction

Two types of nodes in the network, namely herb and symptom, are defined by formulas (1) and (2). In order to construct the overall weighting network, we use the following formula (3) to calculate the edge weight between herb and herb according to the Jaccard index. Formula (4) is applied to calculate the edge weight between symptoms and herbs. The edge weight represents the degree of correlation between nodes.

(1) $$\begin{array}{c} V\left(\text{sa}\right)=\frac{\left|Px\left(\text{sa}\right)\right|}{N}, \end{array}$$

(2) $$\begin{array}{c} V\left(\text{hb}\right)=\frac{\left|Px\left(\text{hb}\right)\right|}{N}, \end{array}$$

(3) $$\begin{array}{c} W\left(\text{hb},\text{hc}\right)=\frac{\left|Px\left(\text{hb}\right)\cap^{}Px\left(\text{hc}\right)\right|}{\left|Px\left(\text{hb}\right)\cap^{}Px\left(hc\right)\right|},\; \end{array}$$

(4) $$\begin{array}{c} W\left(\text{sa},\text{hb}\right)=\frac{\left|Px\left(\text{sa}\right)\cap^{}Px\left(\text{hb}\right)\right|}{Px\left(\text{sa}\right)},\; \end{array}$$

where *V* (sa) represents the symptom node, *V* (hb) represents the herb node, *W* (hb, hc) represents the edge weights between herb and herb, *W* (sa, hb) represents the edge weights between symptom and herb, sa represents symptom *a*, hb represents herb *b*, hc represents herb *c*, *N* represents the number of total prescription, *Px* (hb) represents the prescriptions including the herb *b*, *Px* (hc) represents the prescriptions including the herb *c*, and *Px* (sa) represents the prescriptions including symptom *a*.

### 2.4. Data Statistics and Analysis

#### 2.4.1. Rank of Herb Importance

The PageRank (PR) algorithm has been widely used to identify significant nodes in the network [20–22]. The PageRank score determines the importance of a node based on its entire links. FangNet utilizes the PageRank algorithm to calculate topological hub scores (THScore) about all symptom-to-herb associations in cases, and a segmented linear regression is performed on the calculated THScore to reposition all herbs in the formula [23]. According to the analysis results, driver herbs and passenger herbs can be obtained, in which the former plays a major role in hypertension treatment, while the latter is added or removed only when certain symptoms appear.

#### 2.4.2. Herb-Herb Co-Occurrence Level and Mutual Exclusivity Level

The herb-herb co-occurrence level and mutual exclusivity level were obtained by Fisher's test analysis, based on the herb-herb interaction network. A total of 9 levels were defined, including −1∼4, in which the positive value stood for the co-occurrence level and the higher the value, the more significant the level; while the negative value stood for the mutual exclusivity level and the smaller the value, the more significant the level; and 0 stood for insignificance. The higher co-occurrence level indicates more likely potential interactions between herbs. The analysis results can be visualized through triangular heat maps.

#### 2.4.3. Herb-Symptom Associations

Highly correlated relationships of specific symptoms to herbs were mined based on the edge weights of the symptom-herb network as well as co-occurrence events. We obtained the correlation between herbs and specific symptoms by setting the herb support degree to 0.1∼0.6 and the interaction weight to ≥0.6.

### 2.5. Analysis of Pharmacological Mechanism for Basic Prescriptions

Based on the importance ranking of herbs and co-occurrence level of herbs, we finally obtained a basic prescription consisting of 6 herbs. We searched all the active compounds of these herbs in the TCM systematic pharmacology analysis platform (TCMSP, https://tcmspw.com/tcmsp.php). The candidate compounds were further screened under the conditions of oral bioavailability (OB) ≥30% and drug-like characteristics (DL) ≥0.18 and then transformed into corresponding gene names with the help of UniProt database (https://www.unitprot.org/).

Hypertension was searched as a keyword to obtain protein targets related to hypertension in the GeneCards database (https://www.genecards.org/). Obtained targets with disease were normalized to gene names in the UniProt database.

Venny 2.1 software was used for Venn analysis to obtain the target proteins shared by the candidate active compounds and the disease. Cytoscape 3.6.1 software was used to construct and visualize the herb-compound-target-disease network.

According to the common targets between herbs and diseases, the protein-protein interaction (PPI) network was constructed with the assistance of STRING 11.0 database (https://string-db.org/). All the node information obtained was then imported into Cytoscape 3.6.1 software to determine the core targets based on the degree value. Meanwhile, the main protein functional modules of the PPI network were analyzed through the built-in molecular complex detection (MCODE) plug-in.

With the help of the Database for Annotation Visualization and Integrated Discovery (DAVID, https://david.ncifcrf.gov/), Gene Ontology (GO) function enrichment analysis and the Kyoto Encyclopedia of Genes and Genomes (KEGG) pathway enrichment analysis were performed on target proteins.

### 2.6. Molecular Docking Verification

The significant active compounds in the basic prescription were determined by the degree value in the herb-compound-target-disease network, and the top ten compounds whose degree value exceeded the median were selected as the significant ingredients. For initial validation, we performed molecular docking of core target proteins with important active ingredients and control drugs (valsartan, Betaloc, nifedipine, hydrochlorothiazide, and captopril). MOL2 files of the important active ingredients and control drugs were searched and downloaded from the ZINC database (https://zinc.docking.org/). The PDB format of core target proteins was obtained by searching the RCSB PDB database (https://www.rcsb.org/). To optimize the structures of the core target proteins and avoid the water molecules in them from interfering with the molecular docking results, water molecules of the core target proteins were removed and the original ligands were isolated from the core target proteins with the aid of PyMOL 2.4 software. The targets after hydrogenation, charge calculations, and atomic type setting were saved in the pdbqt format using AutoDock Tools 1.5.6 software. Finally, we ran AutoDock Vina 1.1.2 for molecular docking simulation and visualized the results.

## 3. Results

### 3.1. Rank of Importance of Herb

**Figure 2:**
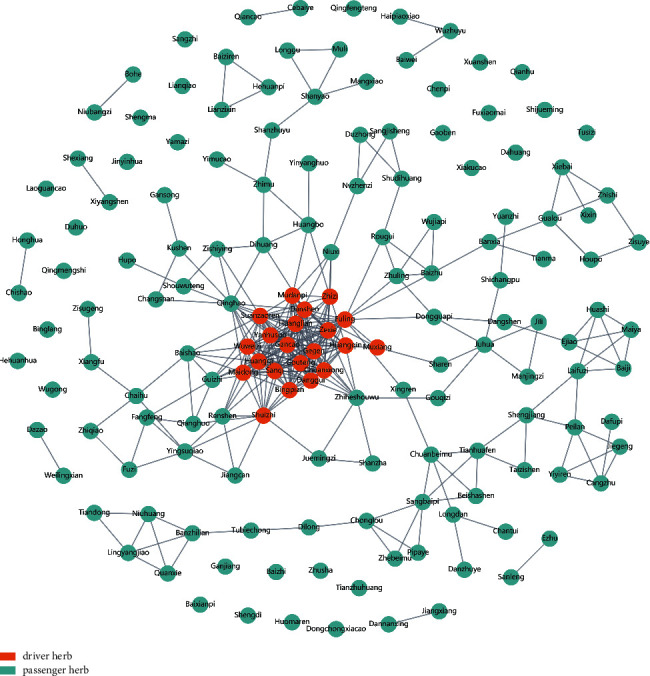
Driver/passenger herb classification. Orange represents driver herbs. Green represents passenger herbs. The edges in the network represent the interactions between herbs.

**Figure 3:**
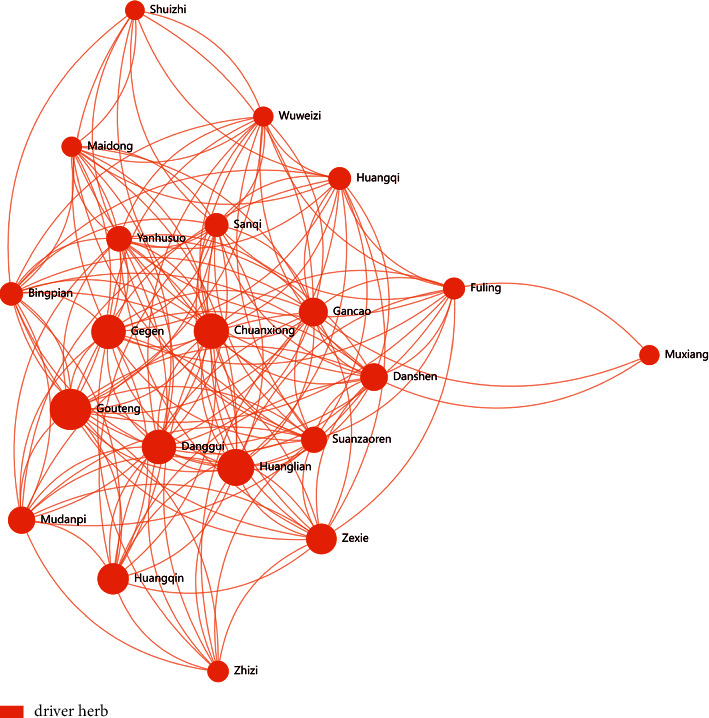
Rank of importance of driver herbs. Orange circle represents driver herbs. The circle size represents THScore.

158 herbs were contained in 439 prescriptions, of which 21 were driver herbs and 137 were passenger herbs. The weight of interactions between herbs was set to ≥0.15, and the interaction relationships were represented in the network in the form of edges, resulting in 344 edges for 158 herbs, as shown in Figure 2. According to THScore, the importance ranking and interaction relationship of driver herbs were extracted, as shown in Figure 3 and Table 1. The higher THScore, the more important the status of related herbs in the treatment of hypertension. As can be seen from the graph, the top six in order of importance are Gouteng (Uncariae Ramulus cum Uncis), Huanglian (Coptidis Rhizoma), Chuanxiong (Chuanxiong Rhizoma), Gegen (Puerariae Lobatae Radix), Danggui (Angelicae Sinensis Radix), and Huangqin (*Scutellariae Radix*). Furthermore, the Latin names, medicinal parts, and efficacy classifications of driver herbs are shown in Table 2.

### 3.2. Co-occurrence and Exclusivity of Herbal Pairs

**Figure 4:**
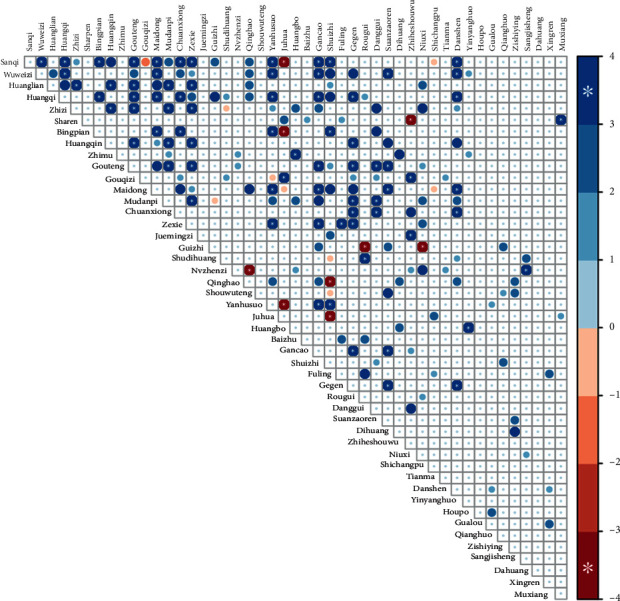
Co-occurrence and exclusivity of herbal pairs.

The triangular heat maps of the co-occurrence and mutual exclusion levels of 47 herbs with frequencies over 0.05 were obtained and are shown in Figure 4. 76 herb pairs with a co-occurrence level of 4 and 9 herb pairs with a mutual exclusivity level of −4 were obtained. The greater the co-occurrence levels and Co_ratio, the more likely the potential interaction between herbs will play a vital role in the disease. The detailed information of the herb pairs with a co-occurrence level of 4, co-occurrence event ≥10, top 10 Co_ratio values, and mutual exclusivity level of −4 was extracted, as shown in Table 3. The co-occurrence levels of Huanglian-Gouteng, Sanqi-Yanhusuo, Huanglian-Huangqin, Chuanxiong-Gegen, Gouteng-Gegen, Wuweizi-Maidong, Gouteng-Danggui, Danggui-Chuanxiong, Huangqi-Maidong, and Gouteng-Huangqin were in the forefront.

### 3.3. Association Analysis of Herbs and Symptoms

**Figure 5:**
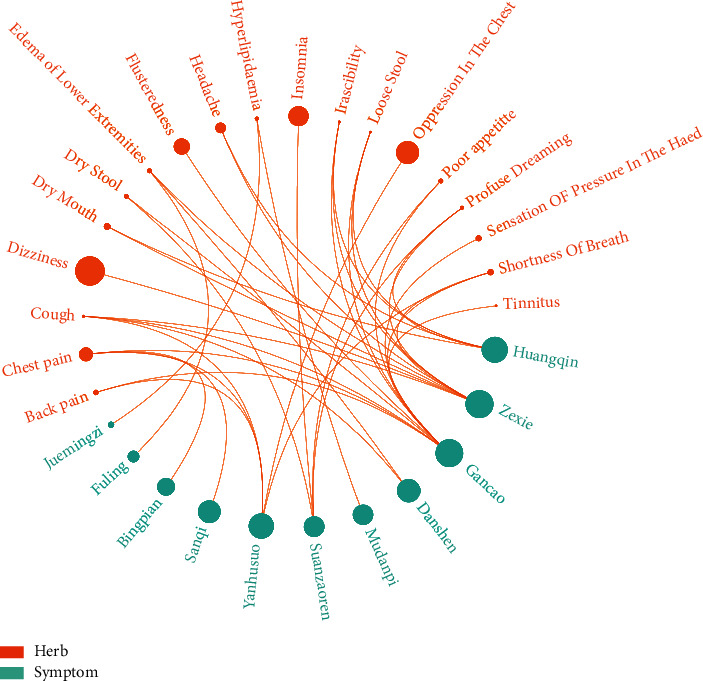
41 high confidence symptom-herb associations. Green represents herbs. Orange represents symptoms. The size of the circle represents herb or symptom support. Edges represent the correlation between herbs and symptoms.

Based on interaction weight ≥0.6 and herbal support degree between 0.1 and 0.6, 41 significant herbs-to-specific-symptoms associations were obtained and visualized in a circular layout, as shown in Figure 5 and Table 4. The symptoms in Table 4 mainly represent the common clinical manifestations or signs of patients with hypertension. As can be seen from the chart, one symptom can be treated by many herbs, and one herb can also treat many symptoms. Therefore, it is of great significance to study the correlation between herbs and symptoms for guiding clinical use of herbs. The higher degree of association included “edema of lower extremities-Zexie (Alismatis Rhizoma), Fuling (Poria); chest pain-Yanhusuo (Corydalis Rhizoma); and loose stool-Zexie (Alismatis Rhizoma).”

### 3.4. Analysis of the Action Mechanism of the Basic Prescription

Based on the herb importance ranking (Table 1), we found 21 driver herbs, of which the top six ranked by importance were Gouteng, Huanglian, Chuanxiong, Gegen, Danggui, and Huangqin, in that order. Referencing to the co-occurrence level of herb pairs (Table 3), strong interaction relationships among Huanglian-Gouteng, Huanglian-Huangqin, Chuanxiong-Gegen, Gouteng-Gegen, Gouteng-Danggui, Danggui-Chuanxiong, and Gouteng-Huangqin were found. Combining Tables 1 and 3, we finally obtained a basic prescription including Gouteng, Huanglian, Chuanxiong, Huangqin, Gegen, and Danggui. These herbs were highly correlated with each other and ranked top in terms of their importance.

A total of 86 candidate active compounds were collected from the TCMSP database after removing duplicate compounds according to the set screening conditions, as detailed in Supplementary Table 1. Among them, 65 compounds were derived from Gouteng, 4 compounds came from Gegen, 4 compounds came from Huanglian, 36 compounds were derived from Huangqin, 7 compounds were derived from Chuanxiong, and 2 compounds came from Danggui. Some compounds were present in more than one herb. Beta-sitosterol was obtained from Gouteng, Gegen, Huangqin, and Danggui. Sitosterol was obtained from Gouteng, Huangqin, and Chuanxiong. Ent-epicatechin was from Gouteng and Huangqin. Quercetin was obtained from Gouteng and Huanglian. Epiberberine and coptisine were obtained from Huanglian and Huangqin. Stigmasterol was obtained from Huangqin and Danggui.

**Figure 6:**
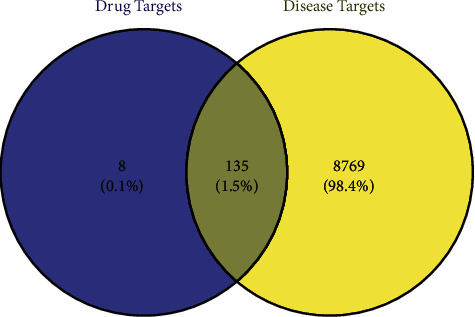
Common targets for herbs and diseases.

8904 disease targets were obtained from the GeneCards database. Using the UniProt database, herb ingredients and disease targets were assigned standardized human gene names. Then, 135 intersection targets of herbs and diseases were found out with the help of the Venn diagram, as shown in Figure 6.

**Figure 7:**
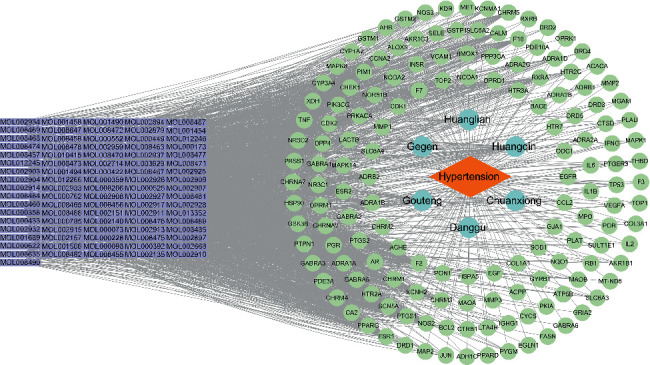
Herb-compound-target-disease network. Blue represents herbs, purple represents compounds, green represents targets, and orange represents hypertension.

The herb-compound-target-disease network was visualized with Cytoscape 3.6.1 software, as shown in Figure 7. Utilizing the “network analyzer” function, the degree values in the network were obtained. The larger the degree value, the more significant the node it represents in the network. The top 10 active compounds whose degree values were greater than the median value (degree values >11) were selected as key ingredients, as shown in Table 5.

**Figure 8:**
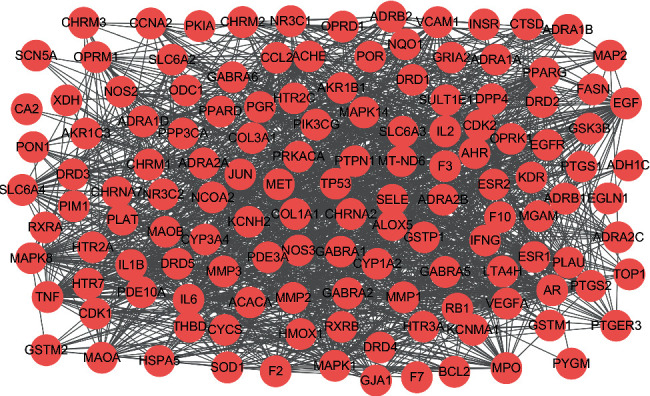
PPI network.

**Figure 9:**
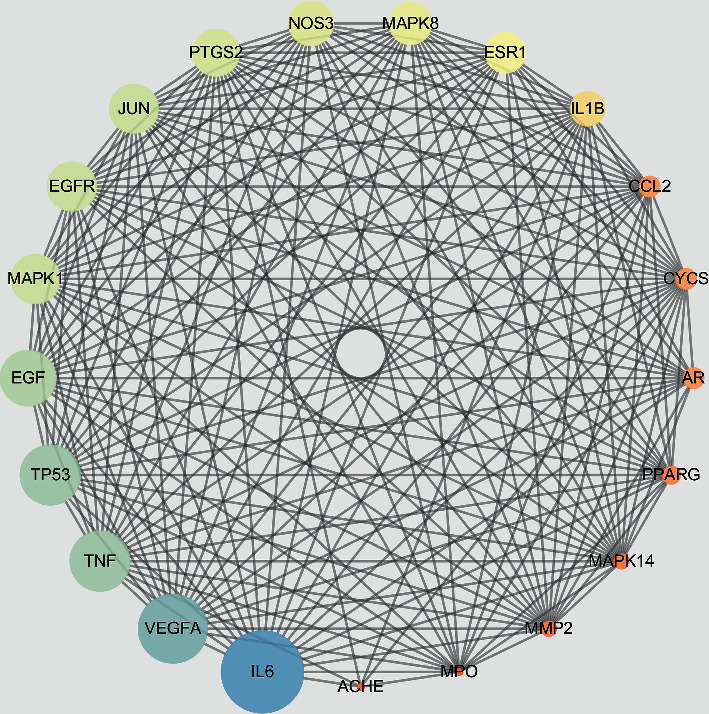
21 core targets.

The intersection target genes were analyzed using the STRING database to construct a PPI network to explore the mechanism of the basic prescription in treating hypertension. After removing free targets, the PPI network with 134 targets and 1409 edges was obtained and visualized in Cytoscape 3.6.1 software, as shown in Figure 8. There were 21 core targets selected based on degree value ≥35, and the results are shown in Figure 9. Among them, IL6, VEGFA, TNF, TP53, and EGF were in the top five order of degree values, and they may hold a more crucial position for hypertension treatment.

**Figure 10:**
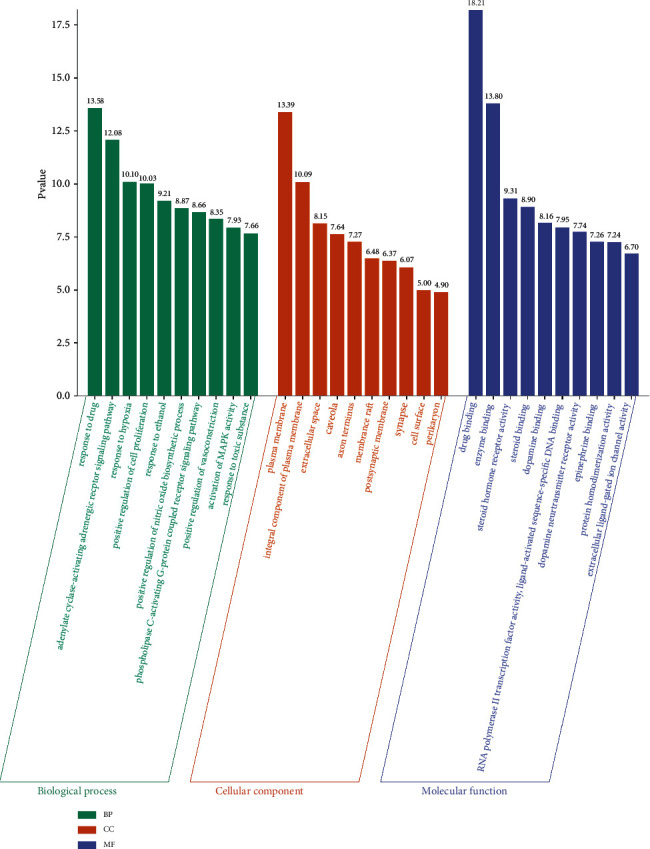
GO pathway analysis (the top 10).

In order to understand the functional role of effective compound targets in the basic prescription more macroscopically and comprehensively, we enriched and analyzed all intersection targets in the DAVID database. The results of GO enrichment analysis included 151 biological processes (BPs), 28 cell components (CCs), and 42 molecular functions (MFs) with a threshold value of *P* < 0.05 and FDR ＜0.05. The top 10 ranked results according to *P* values are shown in Figure 10. The BP results mainly comprised adenylate cyclase-activating adrenergic receptor signaling pathway, response to hypoxia, positive regulation of nitric oxide biosynthetic process, phospholipase C-activating G-protein coupled receptor signaling pathway, positive regulation of vasoconstriction, activation of MAPK activity, and so on. CC analysis showed that the overlapping targets were mainly associated with plasma membrane, integral component of plasma membrane, extracellular space, caveola, axon terminus, membrane raft, and so on. The MF results mainly included drug binding, enzyme binding, steroid hormone receptor activity, epinephrine binding, and extracellular ligand-gated ion channel activity.

**Figure 11:**
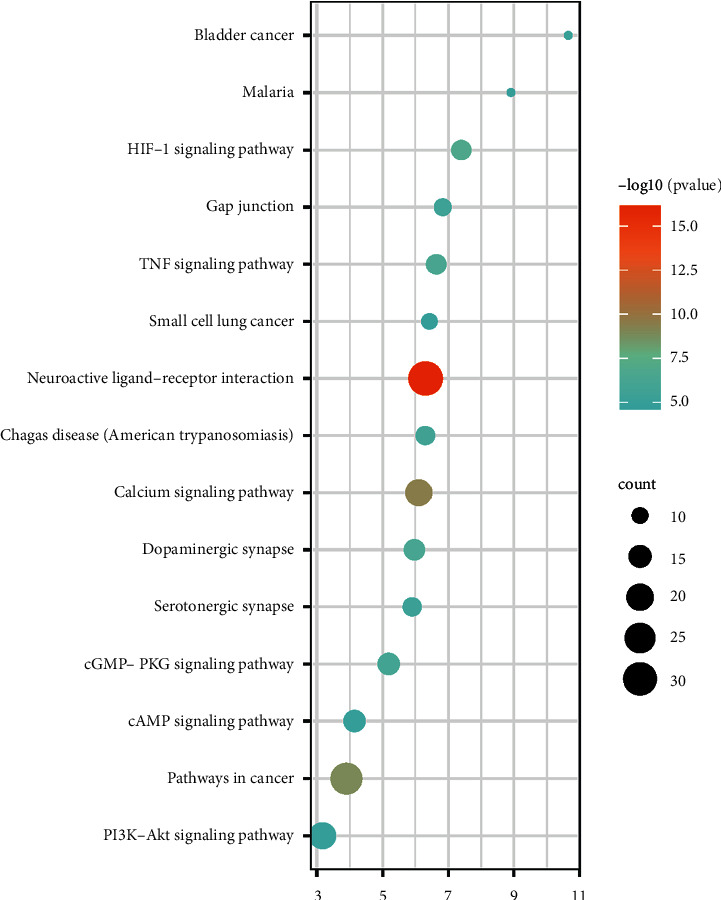
KEGG pathway analysis (the top 15).

KEGG pathway analysis obtained 101 pathways with a threshold value of *P* < 0.05 and FDR ＜0.05, including neuroactive ligand-receptor interaction, calcium signaling pathway, HIF-1 signaling pathway, TNF signaling pathway, cGMP-PKG signaling pathway, cAMP signaling pathway, and PI3K-Akt signaling pathway. The first 15 results of KEGG signal enrichment analysis are shown in Figure 11.

With the in-depth study of systems biology, we found that proteins mostly participate in a common process in the form of protein clusters. Therefore, it was still necessary to further cluster analyze all targets. We used the MCODE plug-in to analyze the PPI network. 7 meaningful protein clusters are shown in Table 6. Among them, 32 main protein targets were contained in the most important cluster 1, containing most core protein targets in Figure 9, which further demonstrated the importance of targets such as IL6, VEGFA, TNF, TP53, and EGF. The full names and acronyms of the targets included in Table 6 are shown in Supplementary Table 2.

**Figure 12:**
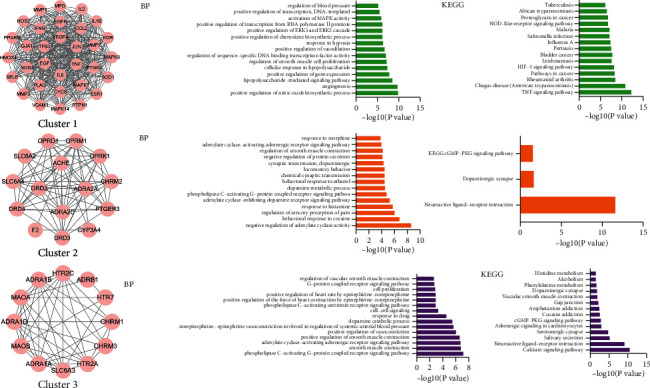
Module analysis of PPI network.

In order to better understand the functions of significant modules and the physiological processes involved, we carried out GO function enrichment analysis and KEGG pathway enrichment analysis for modules with score >4 and carried out visual analysis, as shown in Figure 12. Cluster 1 is associated with antioxidant, anti-inflammatory, and angiogenesis and vascular regulation. Clusters 2 and 3 may regulate blood pressure levels through neuroendocrine pathways. The biological processes related to dopamine and serotonin in Clusters 2 and 3 can also participate in emotion regulation [24]. Related studies have shown that emotion plays an essential role in hypertension [25]. This further reflects the characteristics of simultaneous treatment of mind and body and overall treatment in TCM.

### 3.5. Molecular Docking Validation

**Figure 13:**
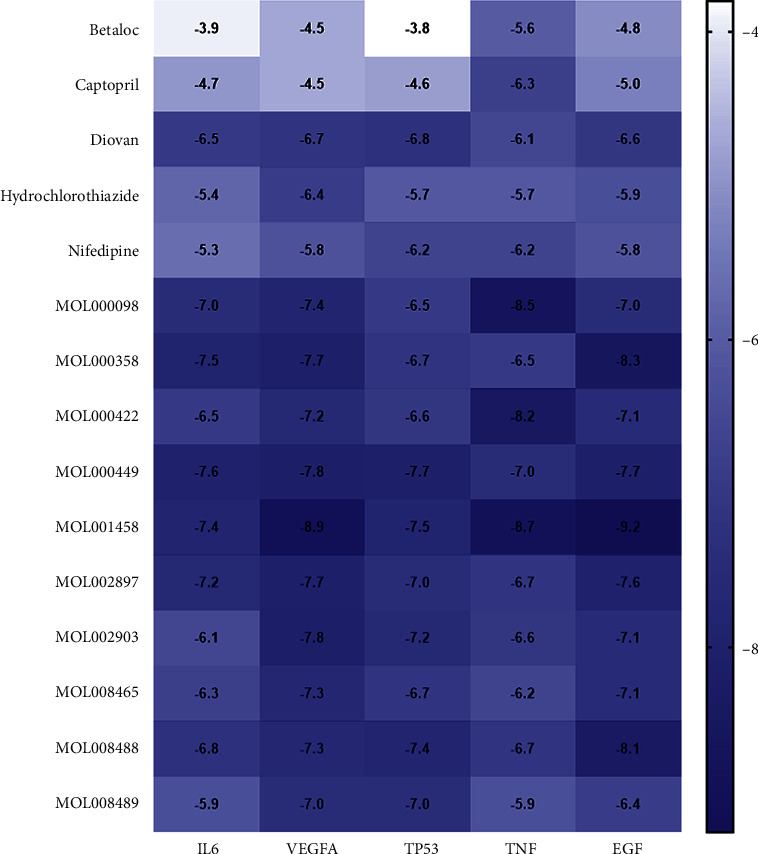
Binding energy of medicines and disease targets.

**Figure 14:**
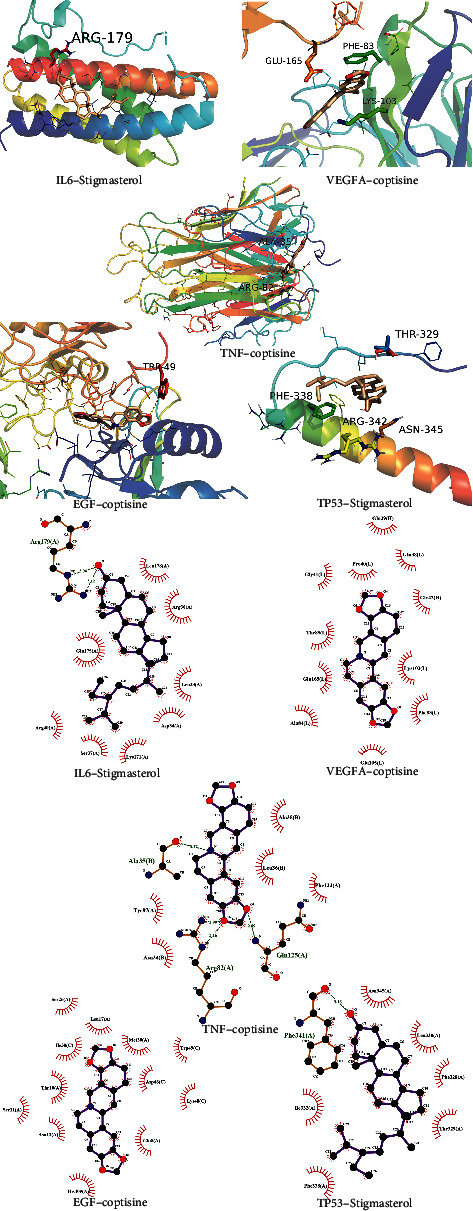
Molecular docking mode.

We selected the top five target proteins (IL6, VEGFA, TNF, TP53, and EGF) in Figure 9 as receptors. Ten significantly active compounds in Table 5 and positive control drugs (valsartan, Betaloc, nifedipine, hydrochlorothiazide, and captopril) were selected as ligands. Using AutoDock software, we performed molecular docking simulation between receptor and ligand, and the results are shown in Figure 13. In general, the binding energy between ligands and receptors is less than zero, which means that they can spontaneously bind, and the lower the binding energy, the stronger the binding ability. In more detail, the most active compound binding to IL6 and TP53 was stigmasterol, whose binding energies were −7.6 and −7.7, respectively. The active compound with the strongest binding ability with VEGFA, TNF, and EGF was coptidine, whose binding energies were −8.9, −8.7, and −9.2, respectively. IL6-stigmasterol, TP53-stigmasterol, VEGFA-coptidine, EGF-coptidine, and TNF-coptidine were used as examples to display 3D and 2D structures, as shown in Figure 14. In summary, the core targets and the active compounds can be tightly combined through hydrogen bonding and hydrophobic interaction, and their overall Vina scores are similar to or higher than those of the positive control drugs and the core targets.

## 4. Discussion

Diseases, symptoms, and herbs are obviously related to each other, rather than independent of each other [26]. Previous studies on TCM data mining only focused on the correlation between herbs and ignored the important role of symptoms in diseases [27]. Judging the importance of herbs in diseases is only based on the frequency of herbs, which is somewhat misleading. Gancao, for instance, tends to be used with high frequency as a harmonizing (blending) drug, but it usually has no sensitivity or specificity for a particular disease or symptom [28]. Additionally, combined herb pairs cannot be ranked and an overview of the weight of each herb cannot be provided. The more critical issue is the failure to make effective use of the association between symptoms and herbs in the analysis of clinical cases. In this study, we innovatively mined the clinical experience of famous TCM doctors in the treatment of hypertension based on structural network algorithm, fully utilized the relationship between symptoms and herbs, defined the importance of herbs in a new way, and obtained detailed quantitative information, which made up for the shortcomings of previous data mining.

Based on the symptom-herb network, three categories of information were obtained. (1) 27 driver herbs: in fact, these herbs have been found to reduce blood pressure [11]. The classification of their functions shows that wind-extinguishing, heat-clearing, dispelling dampness, activating blood circulation, and tonifying are the main treatments for hypertension, which is in accordance with the TCM theory [29]. Interestingly, the support degree of Huanglian was lower than that of Chuanxiong, but its importance was higher than that of Chuanxiong, which may be due to the heat-clearing effect of Huanglian, and heat syndrome appears in various stages of hypertension, especially at the beginning of the disease or before the injury of target organs [30]; (2) 76 pairs of herbs with high co-occurrence level: the higher the co-occurrence level, the greater the possibility of herbaceous-herbaceous interactions. This helps us to understand the composition rule of the prescription and provides ideas for the development of multicomponent new drugs. For example, puerarin extracted from Gegen has been developed as an injection and has been widely used in the treatment of hypertension [31], while Huangqin or Chuanxiong combined with Gegen can promote puerarin absorption in the intestine [32]. In addition, based on (1) and (2), we finally found that Gouteng, Huanglian, Chuanxiong, Gegen, Danggui, and Huangqin were in the forefront of importance and had significant potential interaction relationships with each other. This is considered the basic prescription for the treatment of hypertension; and (3) high confidence relationship between 41 herbs and specific symptoms: the correspondence between specific herbs and personalized symptoms and the correspondence between prescription and syndrome are the basic principle of TCM individualized treatment [33]. These results provide guidance for the personalized clinical application of the basic prescription.

Network pharmacology preliminarily verified the possible pharmacological mechanism of the basic prescription in the treatment of hypertension. Based on the herb-compound-target-disease network, the important active compounds were extracted, which were mainly divided into three categories: flavonoids (quercetin, kaempferol), phytosterols (beta-sitosterol, stigmasterol), and alkaloids (coptisine, epiberberine, (R)-canadine, hirsutine, hirsuteine). Multiple studies have confirmed their beneficial effects on cardiovascular diseases [34–36]. Quercetin and kaempferol have significant antioxidant and anti-inflammatory effects. The study has also suggested that they may be involved in the renin-angiotensin-aldosterone system to control blood pressure by inhibiting angiotensin-converting enzyme (ACE) [37]. Beta-sitosterol and stigmasterol have a wide range of pharmacological effects, such as regulating immunity, anti-inflammatory, and analgesic, regulating blood lipid, inhibiting tumor, and so on [38]. Experimental studies have indicated that beta-sitosterol can regulate arterial relaxation by promoting NO release [39]. Li et al. have demonstrated that stigmasterol can inhibit the proliferation of vascular smooth muscle by mediating apoptosis, so as to maintain the ability of vascular dilation [40]. In addition, multiple alkaloids may play more vital roles. Gong et al. have reported that coptidine induced relaxation of rat aortic rings through endothelium-independent or endothelium-dependent pathways such as the NO/cGMP pathway and activation of potassium channels [41]. Epiberberine has specific antagonistic effects against *β*2-adrenoceptor, which reduces blood pressure by mediating peripheral vasodilation [42]. Yang et al. have confirmed that (R)-canadine could inhibit high-concentration KCl-induced aortic contraction in rats through calcium channel blockers [43]. Huang et al. have discovered a variety of potential vasodilators including hirsutine and hirsuteine based on metabolomics, and animal experiments have revealed that they can dilate the mesenteric artery [44].

Through PPI network analysis, we found five core targets: IL6, VEGFA, TNF, TP53, and EGF, which may play a more important role in the disease. Chronic inflammation, as well as vascular endothelial dysfunction, is closely associated with the occurrence and progression of hypertension and has also increasingly become a research focus of the mechanism against hypertension in recent years. The study has demonstrated that IL6 and TNF, as inflammatory factors, are independent risk factors for hypertension [45]. IL6 can influence the development of Ang II-mediated hypertension by intervening the JAK2/JAK3 pathway [46]. It has been reported that inhibition of TNF-*α* levels can reduce systolic blood pressure and relieve left ventricular hypertrophy and activate the Akt/eNOS pathway to improve vascular endothelial function [47]. VEGFA is a vascular endothelial growth factor that influences angiogenesis and vascular permeability and can also affect blood pressure by promoting nitric oxide (NO)-mediated vasodilation [48]. Clinical studies have found that angiotensin-converting enzyme inhibitors (ACEIs) can lower blood pressure by upregulating VEGF expression [48]. EGF is an epidermal growth factor, which can interfere with epithelial sodium channel to affect renal Na^+^ absorption. It is considered to be a key molecular substrate leading to salt-sensitive hypertension [49]. Moreover, inhibition of epidermal growth factor receptor (EGFR) activity is emerging as a potential treatment for hypertension [50]. Feng et al. have indicated that mir-31a-5p and TP53 were candidate miRNAs and genes regulating hypertension and TP53 was a virtual target gene of mir-31a-5p. Mir-31a-5p was involved in hypertension through accelerated proliferation of arterial smooth muscle cells and inhibition of apoptosis by targeting TP53 [51].

In KEGG pathway analysis, 7 significant pathways closely related to the pathogenesis or treatment of hypertension were found: neuroactive ligand-receptor interaction, calcium signaling pathway, HIF-1 signaling pathway, TNF signaling pathway, cGMP-PKG signaling pathway, cAMP signaling pathway, and PI3K-Akt signaling pathway. Neuroactive ligand-receptor interaction pathways are involved in the regulation of physiological rhythms, endocrine system, mood, learning, and memory [52]. Endocrine disorders and mood disorders are common etiologies leading to hypertension [53, 54]. Selective blocking of calcium influx by acting on the calcium signaling pathway can make blood vessels dilate and reduce vascular resistance [55]. Calcium channel blockers have become one of the main drugs in the treatment of hypertension [56]. The TNF signaling pathway is an important inflammatory signaling pathway, and the study has showed that TNF-*α* may mediate vascular damage in hypertension by inhibiting endothelial cells [57]. Insulin resistance and microcirculatory disturbances are associated with the pathological process of hypertension [58, 59]. Previous studies have documented that activation of the PI3K/AKT pathway can increase endothelial NO production and endothelial NO synthase gene expression to dilate blood vessels and improve microcirculation perfusion in rats [58], but this process could be affected by insulin resistance [60]. Furthermore, HIF-1*α* is regulated by the PI3K/Akt signaling pathway [61]. Upregulation of HIF-1*α* expression is able to protect cardiomyocytes and promote angiogenesis [62]. As ubiquitous second messengers, cGMP and cAMP are widely involved in various cellular functions, such as vasodilation [63]. The cGMP-PKG signaling pathway can lead to vasodilation by activating myosin light chain (MLC) phosphatases [64]. It has been reported that cAMP activates the PKA-CREB pathway to reduce the proliferation and migration of PASMC and PAEC and regulate vascular remodeling [65]. cAMP also participates in the formation of hypertension related osteoporosis by regulating Cbfa1/RANKL pathway [66].

In addition, Gegen, Huanglian, and Huangqin in the basic prescription are the components of Gegen Qinlian decoction, a classic prescription from *Treatise on Cold Damage Disease*. In recent years, there are more and more studies on its treatment of hypertension on China Knowledge Network (https://www.cnki.net/), which further confirms the credibility of the results. However, this study also has obvious limitations: (1) large sample data are the basis of mining potential information. Due to the limitation of the amount of data, the results may be biased; (2) dose is the key to the curative effect of TCM. Although the dose standard deviation of herbs is involved in Table 1, it is of little significance for clinical guidance, so we have not discussed it. How to explore the relationship between herbal dosage and disease or symptoms is the problem that we will focus on in the next step; and (3) although the basic prescription has preliminarily analyzed its pharmacological mechanism through network pharmacology, it is still necessary to carry out in vivo and in vitro tests in the later stage.

## 5. Conclusions

Based on the symptom-herb network, we redefined the important role of herbs in diseases, mined 21 driver herbs, 76 pairs of highly co-occurrence herbs, and 41 herb-symptom associations. Among the driver herbs, the top six are Gouteng, Huanglian, Chuanxiong, Huangqin, and Danggui. In the co-occurrence level of herb pairs, the top 10 significant herb pairs are Huanglian-Gouteng, Sanqi-Yanhusuo, Huanglian-Huangqin, Chuanxiong-Gegen, Gouteng-Gegen, Wuweizi-Maidong, Gouteng-Danggui, Danggui-Chuanxiong, Huangqi-Maidong, and Gouteng-Huangqin. Based on the above results, we summarized the important herb combination composed of Gouteng, Huanglian, Chuanxiong, Gegen, Huangqin, and Danggui as the basic prescription. The basic prescription is involved in anti-inflammation, antioxidation, and protection of vascular endothelium to exert therapeutic effects on hypertension through 10 significant active compounds, 5 core targets, and 7 important pathways. The results indicate that the method adopted in this study can provide an accurate analysis of the prescribing rules of clinicians treating hypertension and effectively mine important herb combinations.

## Figures and Tables

**Table 1:** Rank of importance of driver herbs.

Herb	THScore	Support	Dosage_SD
Gouteng	17.60	0.79	6.85
Huanglian	15.67	0.67	1.42
Chuanxiong	14.87	0.72	2.44
Gegen	14.47	0.70	2.04
Danggui	14.46	0.69	1.32
Huangqin	12.97	0.51	0.40
Zexie	12.62	0.54	4.75
Gancao	11.66	0.54	4.77
Danshen	10.93	0.46	6.95
Mudanpi	10.78	0.40	6.30
Suanzaoren	10.05	0.41	5.50
Yanhusuo	9.87	0.49	4.42
Sanqi	8.85	0.44	1.40
Bingpian	8.54	0.35	0.00
Huangqi	8.22	0.40	9.42
Fuling	7.52	0.23	7.41
Zhizi	7.29	0.28	0.53
Maidong	6.41	0.33	6.73
Muxiang	6.36	0.15	0.00
Wuweizi	6.22	0.29	1.93
Shuizhi	5.93	0.21	4.38

THScore represents network topology hub scores calculated using the PageRank algorithm. Support represents the occurrence frequency of herbs. Dosage_SD represents the standard deviation of different doses of the same herb.

**Table 2:** The Latin names, medicinal parts, and efficacy classifications of driver herbs.

Pinyin	Latin name	Parts used	Herb category
Gouteng	Uncariae Ramulus cum Uncis	Stem branch	Liver-pacifying and wind-extinguishing
Huanglian	Coptidis Rhizoma	Rhizome	Heat-clearing
Chuanxiong	Chuanxiong Rhizoma	Rhizome	Blooding-activating and stasis-resolving
Gegen	Puerariae Lobatae Radix	Root	Exterior-releasing
Danggui	Angelicae Sinensis Radix	Root	Tonifying
Huangqin	Scutellariae Radix	Root	Heat-clearing
Zexie	Alismatis Rhizoma	Tuber	Dampness-draining diuretic
Gancao	Glycyrrhizae Radix et Rhizoma	Root and rhizome	Tonifying
Danshen	Salviae Miltiorrhizae Radix et Rhizoma	Root and rhizome	Blooding-activating and stasis-resolving
Mudanpi	Moutan Cortex	Root bark	Heat-clearing
Suanzaoren	Ziziphi Spinosae Semen	Fruit	Tranquilizing
Yanhusuo	Corydalis Rhizoma	Tuber	Blooding-activating and stasis-resolving
Sanqi	Notoginseng Radix et Rhizoma	Root and rhizome	Stasis-resolving hemostatic
Bingpian	Borneolum Syntheticum	Resin processed products	Resuscitation
Huangqi	Astragali radix	Root	Tonifying
Fuling	Poria	Sclerotium	Dampness-draining diuretic
Zhizi	Gardeniae Fructus	Fruit	Heat-clearing
Maidong	Ophiopogonis Radix	Root	Tonifying
Muxiang	Aucklandiae Radix	Root	Qi-regulating
Wuweizi	Schisandrae Chinensis Fructus	Fruit	Astringent
Shuizhi	Hirudo	Whole	Blooding-activating and stasis-resolving

**Table 3:** The herb pairs with high co-occurrence level and high mutual exclusivity level.

Herb1	Herb2	*P*value	Co_ratio	Co_event	Ex_event	Total_event	Co_level
Huanglian	Gouteng	0.001	0.75	274	91	365	4
Sanqi	Yanhusuo	0.001	0.72	173	66	239	4
Huanglian	Huangqin	0.001	0.72	215	84	299	4
Chuanxiong	Gegen	0.001	0.7	257	109	366	4
Gouteng	Gegen	0.001	0.7	269	118	387	4
Wuweizi	Maidong	0.001	0.67	109	53	162	4
Gouteng	Danggui	0.001	0.66	259	133	392	4
Danggui	Chuanxiong	0.001	0.63	240	138	378	4
Huangqi	Maidong	0.001	0.63	125	72	197	4
Gouteng	Huangqin	0.001	0.63	219	131	350	4
Sharen	Heshouwu	0.001	0	0	120	120	−4
Rougui	Guizhi	0.008	0	0	93	93	−4
Qinghao	Shuizhi	0.001	0	0	143	143	−4
Nuzhenzi	Qinghao	0.009	0	0	91	91	−4
Niuxi	Guizhi	0.001	0	0	110	110	−4
Juhua	Yanhusuo	0.001	0	0	242	242	−4
Juhua	Sanqi	0.001	0	0	220	220	−4
Juhua	Bingpian	0.001	0	0	177	177	−4
Juhua	Shuizhi	0.004	0	0	118	118	−4

*P* values were calculated by Fisher's test. Co_event represents the sum of the number of times two herbs appeared together in the same prescription. Ex_event represents the number of times either of the two herbs appeared in the prescription. Total_Event represents the sum of Co_event and Ex_event. Co_ratio represents Co_event/Total_event. Co_level represents the level of co-occurrence and mutual exclusion.

**Table 4:** 41 significant associations between symptoms and herbs.

Symptom	Herb	Association	Symptom_support	Herb_support
Edema of lower extremities	Zexie	0.89	0.1	0.54
	Fuling	0.8	0.1	0.23
	Danshen	0.68	0.1	0.46

Chest pain	Yanhusuo	0.86	0.28	0.49
	Sanqi	0.72	0.28	0.44
	Bingpian	0.67	0.28	0.35
	Gancao	0.64	0.28	0.54

Loose stool	Zexie	0.8	0.06	0.54
	Gancao	0.68	0.06	0.54
	Huangqin	0.68	0.06	0.51

Hyperlipidemia	Juemingzi	0.79	0.09	0.13
	Mudanpi	0.63	0.09	0.4

Profuse dreaming	Suanzaoren	0.72	0.08	0.41
	Huangqin	0.67	0.08	0.51
	Zexie	0.61	0.08	0.54

Cough	Danshen	0.71	0.06	0.46
	Gancao	0.68	0.06	0.54
	Zexie	0.61	0.06	0.54
	Yanhusuo	0.61	0.06	0.49

Dry mouth	Zexie	0.7	0.14	0.54
	Huangqin	0.69	0.14	0.51

Back pain	Gancao	0.69	0.11	0.54
	Yanhusuo	0.65	0.11	0.49

Poor appetite	Gancao	0.68	0.1	0.54
	Suanzaoren	0.61	0.1	0.41

Shortness of breath	Yanhusuo	0.66	0.13	0.49
	Zexie	0.64	0.13	0.54
	Gancao	0.64	0.13	0.54

Irascibility	Gancao	0.63	0.06	0.54
	Zexie	0.63	0.06	0.54
	Huangqin	0.63	0.06	0.51

Dry stool	Gancao	0.65	0.1	0.54
	Suanzaoren	0.6	0.1	0.41

Headache	Huangqin	0.63	0.21	0.51
	Zexie	0.6	0.21	0.54

Insomnia	Suanzaoren	0.68	0.39	0.41
Oppression in the chest	Yanhusuo	0.65	0.45	0.49
Sensation of pressure in the head	Gancao	0.65	0.12	0.54
Tinnitus	Gancao	0.64	0.06	0.54
Dizziness	Zexie	0.61	0.57	0.54
Flusteredness	Gancao	0.61	0.32	0.54

**Table 5:** The top 10 active compounds.

Mol Id	Molecule name	Degree	Betweenness centrality	Closeness centrality
MOL000358	Beta-sitosterol	208	0.048	0.449
MOL000098	Quercetin	176	0.145	0.512
MOL000449	Stigmasterol	90	0.042	0.433
MOL000422	Kaempferol	56	0.039	0.444
MOL002903	(R)-canadine	49	0.019	0.435
MOL002897	Epiberberine	46	0.004	0.404
MOL008488	Yohimbine	45	0.014	0.426
MOL008489	Hirsuteine	43	0.010	0.423
MOL001458	Coptisine	42	0.003	0.402
MOL008465	Hirsutine	42	0.017	0.421

**Table 6:** MCODE network topology analysis results.

Cluster	Score	Nodes	Edges	Targets
1	26.258	32	407	ESR1, TNF, PTPN1, PTGS2, MAPK1, MMP2, MAPK14, CYCS, IL6, VCAM1, PLAU, NOS3, GJA1, SELE, IFNG, MMP3, EGF, EGFR, HMOX1, CCL2, PPARG, IL2, MAPK8, JUN, NOS2, MMP1, MPO, TP53, IL1B, KDR, SOD1, VEGFA
2	10.286	15	72	SLC6A2, ADRA2A, ADRA2C, OPRM1, OPRD1, OPRK1, CHRM2, PTGER3, CYP3A4, DRD3, DRD2, F2, ACHE, DRD4, SLC6A4
3	8	12	44	SLC6A3, CHRM3, HTR2C, CHRM1, ADRA1D, ADRA1A, HTR7, ADRA1B, ADRB1, MAOA, HTR2A, MAOB
4	4	5	8	AHR, NR3C1, GSK3B, PGR, AR
5	4	4	6	CDK1, CCNA2, RB1, CDK2
6	3	3	3	DRD1, DRD5, HTR3A
7	3	3	3	GABRA6, GABRA5, GABRA1

## Data Availability

All the data used to support the findings of this study are available from the corresponding author upon reasonable request.

## Supplementary Materials

Supplementary Materials Table S1. The 86 candidate active compounds screened from TCMSP database. Table S2. The full names and acronyms of targets in Table 6.
