# Supplementary material for: Mining Important Herb Combinations of Traditional Chinese Medicine against Hypertension Based on the Symptom-Herb Network Combined with Network Pharmacology
Source: Evid Based Complement Alternat Med. 2022 Mar 22;2022:5850899. doi: 10.1155/2022/5850899 (PMC8964163; doi:10.1155/2022/5850899)
Supplement: Supplementary Materials — Table S1. The 86 candidate active compounds screened from TCMSP database. Table S2. The full names and acronyms of targets in Table 6. [file 5850899.f1.zip › 5850899.f1/Table S1. The 86 candidate active compounds screened from TCMSP database.docx]

Table S1. The 86 candidate active compounds screened from TCMSP database

| Mol ID | Molecule Name | OB (%) | DL |
| --- | --- | --- | --- |
| MOL000358 | beta-sitosterol | 36.91 | 0.75 |
| MOL000359 | sitosterol | 36.91 | 0.75 |
| MOL000422 | kaempferol | 41.88 | 0.24 |
| MOL000073 | ent-Epicatechin | 48.96 | 0.24 |
| MOL008455 | 3-oxo-22α-hydroxyurs-12-en-27,28-dioc acid | 32.33 | 0.68 |
| MOL008456 | (3E,4R)-4-(1,3-benzodioxol-5-ylmethyl)-3-[(3,4,5-trimethoxyphenyl)methylidene]oxolan-2-one | 51.78 | 0.65 |
| MOL008457 | Tetrahydroalstonine | 32.42 | 0.81 |
| MOL008458 | Angustidine | 51.85 | 0.66 |
| MOL008460 | geissoschizinc acid | 49.92 | 0.60 |
| MOL008463 | SMR000232338 | 56.74 | 0.75 |
| MOL008465 | hirsutine | 32.75 | 0.64 |
| MOL008467 | Rhynchophylline A | 68.68 | 0.69 |
| MOL008468 | methyl (E)-2-[(2S,3Z,12bS)-3-ethylidene-2,4,6,7,12,12b-hexahydro-1H-indolo[3,2-h]quinolizin-2-yl]-3-methoxyprop-2-enoate | 56.83 | 0.64 |
| MOL008469 | Rhynchophylline | 41.82 | 0.57 |
| MOL008470 | SMR000232333 | 78.38 | 0.75 |
| MOL008471 | Isorhyncophylline | 47.31 | 0.57 |
| MOL008472 | hirsutasideA | 70.34 | 0.81 |
| MOL008473 | (E)-2-[(3S,6'S,7'S,8'aS)-6'-ethyl-2-keto-spiro[indoline-3,1'-indolizidine]-7'-yl]-3-methoxy-acrylic acid methyl ester | 57.85 | 0.57 |
| MOL008474 | (E)-2-[(3R,6'S,7'S,8'aS)-6'-ethyl-2-keto-spiro[indoline-3,1'-indolizidine]-7'-yl]-3-methoxy-acrylic acid methyl ester | 54.47 | 0.57 |
| MOL008475 | Mitraphyllic acid | 31.70 | 0.70 |
| MOL008476 | hirsutasideB | 40.21 | 0.80 |
| MOL008477 | corynoxeine | 57.13 | 0.57 |
| MOL008478 | methyl (E)-2-[(2S,3R,12bS)-3-vinyl-1,2,3,4,6,7,12,12b-octahydroindolo[3,2-h]quinolizin-2-yl]-3-methoxy-prop-2-enoate | 31.94 | 0.64 |
| MOL008481 | (1'R,3S,4a'S,5a'S,10a'R)-1'-methyl-2-oxo-1',4a',5',5a',7',8',10',10a'-octahydrospiro[indoline-3,6'-pyrano[3,4-f]indolizine]-4'-carboxylic acid | 105.22 | 0.70 |
| MOL008482 | (2S,12bR)-methyl 2-((E)-1-oxobut-2-en-2-yl)-1,2,6,7,12,12b-hexahydroindolo[2,3-a] quinolizine-3-carboxylate | 42.07 | 0.60 |
| MOL008484 | vincoside lactam_qt | 50.81 | 0.82 |
| MOL008485 | hirsutasideC | 34.27 | 0.75 |
| MOL008487 | (E)-2-[(2S,3R,12bR)-3-ethyl-1,2,3,4,6,7,12,12b-octahydroindolo[2,3-a]quinolizin-2-yl]-3-methoxyprop-2-enoate | 34.44 | 0.43 |
| MOL008488 | yohimbine | 46.42 | 0.81 |
| MOL008489 | hirsuteine | 41.64 | 0.64 |
| MOL008490 | isocorynantheic acid | 72.36 | 0.60 |
| MOL000098 | quercetin | 46.43 | 0.28 |
| MOL008635 | coryincine | 38.27 | 0.81 |
| MOL000392 | formononetin | 69.67 | 0.21 |
| MOL002959 | 3'-Methoxydaidzein | 48.57 | 0.24 |
| MOL003629 | Daidzein-4,7-diglucoside | 47.27 | 0.67 |
| MOL001454 | berberine | 36.86 | 0.78 |
| MOL013352 | Obacunone | 43.29 | 0.77 |
| MOL002894 | berberrubine | 35.74 | 0.73 |
| MOL002897 | epiberberine | 43.09 | 0.78 |
| MOL002903 | (R)-Canadine | 55.37 | 0.77 |
| MOL002904 | Berlambine | 36.68 | 0.82 |
| MOL002907 | Corchoroside A_qt | 104.95 | 0.78 |
| MOL000622 | Magnograndiolide | 63.71 | 0.19 |
| MOL000762 | Palmidin A | 35.36 | 0.65 |
| MOL000785 | palmatine | 64.60 | 0.65 |
| MOL001458 | coptisine | 30.67 | 0.86 |
| MOL002668 | Worenine | 45.83 | 0.87 |
| MOL008647 | Moupinamide | 86.71 | 0.26 |
| MOL001689 | acacetin | 34.97 | 0.24 |
| MOL000173 | wogonin | 30.68 | 0.23 |
| MOL000228 | (2R)-7-hydroxy-5-methoxy-2-phenylchroman-4-one | 55.23 | 0.20 |
| MOL002714 | baicalein | 33.52 | 0.21 |
| MOL002908 | 5,8,2'-Trihydroxy-7-methoxyflavone | 37.01 | 0.27 |
| MOL002909 | 5,7,2,5-tetrahydroxy-8,6-dimethoxyflavone | 33.82 | 0.45 |
| MOL002910 | Carthamidin | 41.15 | 0.24 |
| MOL002911 | 2,6,2',4'-tetrahydroxy-6'-methoxychaleone | 69.04 | 0.22 |
| MOL002913 | Dihydrobaicalin_qt | 40.04 | 0.21 |
| MOL002914 | Eriodyctiol (flavanone) | 41.35 | 0.24 |
| MOL002915 | Salvigenin | 49.07 | 0.33 |
| MOL002917 | 5,2',6'-Trihydroxy-7,8-dimethoxyflavone | 45.05 | 0.33 |
| MOL002925 | 5,7,2',6'-Tetrahydroxyflavone | 37.01 | 0.24 |
| MOL002926 | dihydrooroxylin A | 38.72 | 0.23 |
| MOL002927 | Skullcapflavone II | 69.51 | 0.44 |
| MOL002928 | oroxylin a | 41.37 | 0.23 |
| MOL002932 | Panicolin | 76.26 | 0.29 |
| MOL002933 | 5,7,4'-Trihydroxy-8-methoxyflavone | 36.56 | 0.27 |
| MOL002934 | NEOBAICALEIN | 104.34 | 0.44 |
| MOL002937 | DIHYDROOROXYLIN | 66.06 | 0.23 |
| MOL000525 | Norwogonin | 39.40 | 0.21 |
| MOL000552 | 5,2'-Dihydroxy-6,7,8-trimethoxyflavone | 31.71 | 0.35 |
| MOL000449 | Stigmasterol | 43.83 | 0.76 |
| MOL001490 | bis[(2S)-2-ethylhexyl] benzene-1,2-dicarboxylate | 43.59 | 0.35 |
| MOL001506 | Supraene | 33.55 | 0.42 |
| MOL002879 | Diop | 43.59 | 0.39 |
| MOL008206 | Moslosooflavone | 44.09 | 0.25 |
| MOL010415 | 11,13-Eicosadienoic acid, methyl ester | 39.28 | 0.23 |
| MOL012245 | 5,7,4'-trihydroxy-6-methoxyflavanone | 36.63 | 0.27 |
| MOL012246 | 5,7,4'-trihydroxy-8-methoxyflavanone | 74.24 | 0.26 |
| MOL012266 | rivularin | 37.94 | 0.37 |
| MOL001494 | Mandenol | 42.00 | 0.19 |
| MOL002135 | Myricanone | 40.60 | 0.51 |
| MOL002140 | Perlolyrine | 65.95 | 0.27 |
| MOL002151 | senkyunone | 47.66 | 0.24 |
| MOL002157 | wallichilide | 42.31 | 0.71 |
| MOL000433 | FA | 68.96 | 0.71 |
