# Supplementary material for: Mining Important Herb Combinations of Traditional Chinese Medicine against Hypertension Based on the Symptom-Herb Network Combined with Network Pharmacology
Source: Evid Based Complement Alternat Med. 2022 Mar 22;2022:5850899. doi: 10.1155/2022/5850899 (PMC8964163; doi:10.1155/2022/5850899)
Supplement: Supplementary Materials — Table S1. The 86 candidate active compounds screened from TCMSP database. Table S2. The full names and acronyms of targets in Table 6. [file 5850899.f1.zip › 5850899.f1/Table S2. The full names and acronyms of targets in Table 6 (1).docx]

Table S2. The full names and acronyms of targets in Table 6

| Cluster | Full Name | Acronyms |
| --- | --- | --- |
| 1 | Estrogen Receptor 1 | ESR1 |
| 1 | Tumor Necrosis Factor | TNF |
| 1 | Protein Tyrosine Phosphatase Non-Receptor Type 1 | PTPN1 |
| 1 | Prostaglandin-Endoperoxide Synthase 2 | PTGS2 |
| 1 | Mitogen-Activated Protein Kinase 1 | MAPK1 |
| 1 | Matrix Metallopeptidase 2 | MMP2 |
| 1 | Mitogen-Activated Protein Kinase 14 | MAPK14 |
| 1 | Cytochrome C, Somatic | CYCS |
| 1 | Interleukin 6 | IL6 |
| 1 | Vascular Cell Adhesion Molecule 1 | VCAM1 |
| 1 | Plasminogen Activator, Urokinase | PLAU |
| 1 | Nitric Oxide Synthase 3 | NOS3 |
| 1 | Gap Junction Protein Alpha 1 | GJA1 |
| 1 | Selectin E | SELE |
| 1 | Interferon Gamma | IFNG |
| 1 | Matrix Metallopeptidase 3 | MMP3 |
| 1 | Epidermal Growth Factor | EGF |
| 1 | Epidermal Growth Factor Receptor | EGFR |
| 1 | Heme Oxygenase 1 | HMOX1 |
| 1 | C-C Motif Chemokine Ligand 2 | CCL2 |
| 1 | Peroxisome Proliferator Activated Receptor Gamma | PPARG |
| 1 | Interleukin 2 | IL2 |
| 1 | Mitogen-Activated Protein Kinase 8 | MAPK8 |
| 1 | Jun Proto-Oncogene, AP-1 Transcription Factor Subunit | JUN |
| 1 | Nitric Oxide Synthase 2 | NOS2 |
| 1 | Matrix Metallopeptidase 1 | MMP1 |
| 1 | Myeloperoxidase | MPO |
| 1 | Tumor Protein P53 | TP53 |
| 1 | Interleukin 1 Beta | IL1B |
| 1 | Kinase Insert Domain Receptor | KDR |
| 1 | Superoxide Dismutase 1 | SOD1 |
| 1 | Vascular Endothelial Growth Factor A | VEGFA |
| 2 | Solute Carrier Family 6 Member 2 | SLC6A2 |
| 2 | Adrenoceptor Alpha 2A | ADRA2A |
| 2 | Adrenoceptor Alpha 2C | ADRA2C |
| 2 | Opioid Receptor Mu 1 | OPRM1 |
| 2 | Opioid Receptor Delta 1 | OPRD1 |
| 2 | Opioid Receptor Kappa 1 | OPRK1 |
| 2 | Cholinergic Receptor Muscarinic 2 | CHRM2 |
| 2 | Prostaglandin E Receptor 3 | PTGER3 |
| 2 | Cytochrome P450 Family 3 Subfamily A Member 4 | CYP3A4 |
| 2 | Dopamine Receptor D3 | DRD3 |
| 2 | Dopamine Receptor D2 | DRD2 |
| 2 | Coagulation Factor II, Thrombin | F2 |
| 2 | Acetylcholinesterase (Cartwright Blood Group) | ACHE |
| 2 | Dopamine Receptor D4 | DRD4 |
| 2 | Solute Carrier Family 6 Member 4 | SLC6A4 |
| 3 | Solute Carrier Family 6 Member 3 | SLC6A3 |
| 3 | Cholinergic Receptor Muscarinic 3 | CHRM3 |
| 3 | 5-Hydroxytryptamine Receptor 2C | HTR2C |
| 3 | Cholinergic Receptor Muscarinic 1 | CHRM1 |
| 3 | Adrenoceptor Alpha 1D | ADRA1D |
| 3 | Adrenoceptor Alpha 1A | ADRA1A |
| 3 | 5-Hydroxytryptamine Receptor 7 | HTR7 |
| 3 | Adrenoceptor Alpha 1B | ADRA1B |
| 3 | Adrenoceptor Beta 1 | ADRB1 |
| 3 | Monoamine Oxidase A | MAOA |
| 3 | 5-Hydroxytryptamine Receptor 2A | HTR2A |
| 3 | Monoamine Oxidase B | MAOB |
| 4 | Aryl Hydrocarbon Receptor | AHR |
| 4 | Nuclear Receptor Subfamily 3 Group C Member 1 | NR3C1 |
| 4 | Glycogen Synthase Kinase 3 Beta | GSK3B |
| 4 | Progesterone Receptor | PGR |
| 4 | Androgen Receptor | AR |
| 5 | Cyclin Dependent Kinase 1 | CDK1 |
| 5 | Cyclin A2 | CCNA2 |
| 5 | RB Transcriptional Corepressor 1 | RB1 |
| 5 | Cyclin Dependent Kinase 2 | CDK2 |
| 6 | Dopamine Receptor D1 | DRD1 |
| 6 | Dopamine Receptor D5 | DRD5 |
| 6 | 5-Hydroxytryptamine Receptor 3A | HTR3A |
| 7 | Gamma-Aminobutyric Acid Type A Receptor Subunit Alpha6 | GABRA6 |
| 7 | Gamma-Aminobutyric Acid Type A Receptor Subunit Alpha5 | GABRA5 |
| 7 | Gamma-Aminobutyric Acid Type A Receptor Subunit Alpha1 | GABRA1 |
